# Effectiveness of acupuncture for irritable bowel syndrome: Protocol for a scoping review of systematic reviews and meta-analyses

**DOI:** 10.1097/MD.0000000000029218

**Published:** 2022-07-22

**Authors:** Yachen Li, Sike Peng, Fangyuan Liang, Suzhen Liu, Jia Li

**Affiliations:** College of Acupuncture and Orthopedics, Hubei University of Chinese Medicine, Wuhan, China.

**Keywords:** acupuncture, irritable bowel syndrome, meta-analyses, scoping review, systematic reviews

## Abstract

Irritable bowel syndrome (IBS) is a common functional bowel disorder. Many systematic reviews/meta-analyses indicate that acupuncture and related therapies are effective for IBS. However, the robustness of the results in the systematic reviews and meta-analyses has not been evaluated. This scoping review aims to ascertain the credibility of current evidence of acupuncture therapy for IBS, to provide clinical research investigators with reliable information.

**Methods:**

Searches of China National Knowledge Infrastructure (CNKI), China Science and Technology Journal Database (VIP), China Biology Medicine disc (CBMdisc), and Wanfang Database since the establishment of the database to February 2022. Study selection and data extraction will be conducted by 2 reviewers, and the quality will be assessed by 2 trained reviewers. We will use Assessment of Multiple Systematic Reviews-2 (AMSTAR2) for methodological quality assessment, Preferred Reporting Items for Systematic Reviews and Meta-Analyses for report quality assessment, Grading of Recommendations, Assessment, Development, and Evaluation for the quality of evidence assessment, and the Risk of Bias in Systematic Reviews for the bias assessment.

**Results:**

The results will be published in a peer-reviewed journal.

**Trial registration number:**

INPLASY202210117.

**Conclusion:**

This scoping review will provide comprehensive evidence of acupuncture for patients with irritable bowel syndrome.

**Ethics and dissemination:**

This scoping review does not require ethical approval as it is a secondary assessment of available literature.

## 1. Introduction

Irritable bowel syndrome (IBS), a common functional bowel disorder, is characterized by recurrent abdominal pain and abdominal distention that include defecation or a change in bowel habits.^[1]^ According to the latest Rome criteria (Rome IV),^[2]^ IBS is classified into IBS with constipation (IBC-C); IBS with diarrhea (IBS-D); IBS with mixed symptomology (IBS-M); and unclassified IBS. According to the statistics, IBS affects about 11% of the world's population.^[3]^ IBS is a common but not life-threatening disease with recurrent symptoms and a chronic course. It significantly affects the quality of life, work productivity, and leads to economic loss,^[4,5]^ imposing a great socioeconomic burden.^[6,7]^

The etiology of IBS is very complicated, which can be caused by various factors, including visceral hyperalgesia, inflammatory, genetic, gastrointestinal motility disorder, intestinal infection, and psychosocial factors.^[8]^ Additionally, the exact pathogenesis for IBS remains unclear. There are currently no clinical symptoms that can be explained by definite organic or biochemical abnormalities^[9,10]^ Therefore, most of the current common therapies are aimed at symptom management rather than disease modification.^[11]^ The first-line treatments include lifestyle changes, psychological treatment, specialized diets, and antispasmodic drugs.^[12]^ For patients with different symptoms and severity, visceral analgesics, prokinetic, antidiarrheal, antidepressants, and psychological interventions are often used clinically.^[13]^ Although these interventions can relieve symptoms, there are still many side effects that patients are not satisfied with.^[14]^ Additionally, pharmacotherapies may increase the odds of cardiovascular events and ischemic colitis.^[12]^

It has gradually become a challenge for scholars in the field of digestion to seek a Comprehensive and alternative medicine (CAM).^[15]^ Acupuncture, a component of traditional Chinese medicine, is also one of the most widely used forms of CAM.^[16]^ Acupuncture treats diseases by stimulating certain acupoints to dredge the meridians and collaterals and to harmonize viscera, qi, and blood.^[17]^ In recent years, many clinical and experimental studies in domestic and foreign shown that acupuncture is effective in the treatment of IBS.^[18–20]^ It can not only effectively improve clinical symptoms and decrease recurrence, but also avoid the adverse effects of western medicine. Acupuncture can play a pivotal role in regulating gastrointestinal motility, inhibiting visceral hypersensitivity, affecting brain-gut axis, regulating immunity and the intestinal flora. As a non-pharmacological treatment method, acupuncture is characteristic as safe, effective, and economic-friendly compared with other approaches.^[15]^ Thus, acupuncture and intervention treatment of IBS have been used widely and paid more and more attention.

Numerous randomized controlled studies (RCTs) have been conducted to investigate the effectiveness and safety of acupuncture in the treatment of IBS.^[2,21,22]^ With the development of clinical evidence-based medicine, relevant systematic reviews (SRs) and meta-analyses (MAs) have been published continuously.^[23–25]^ As we all know, SRs/MAs are significant sources of the best evidence, which could provide evidence-based and reliable conclusions for clinical decision-making. On the other hand, low-quality studies not only tend to mislead researchers and doctors and affect further research but also seriously weaken the validity of research results. Only high-quality SRs and MAs can give clinicians and clinical research investigators reliable data and research directions for future studies. Scoping reviews of SR and MAs can give compiled evidence as well as synthesize the results of many SRs and MAs.^[26]^ Nevertheless, no critically designed scoping review has been carried out so far. This study will conduct a comprehensive evaluation of the methodological and reporting quality of the included SRs/MAs, as well as the evidence quality. The results of this scoping review will provide patients and clinical research investigators with the credibility of current evidence of acupuncture therapy for IBS.

## 2. Methods

### 2.1. Study registration

Our protocol of scoping review was designed according to the guideline of the Preferred Reporting Items for Systematic Reviews and Meta-analyses Protocols (PRISMA-P).^[27]^ It is registered in INPLASY (202210117; DOI: 10.37766/inplasy2022.1.0117).

### 2.2. Criteria for inclusion of studies

#### 2.2.1. Types of studies.

SRs and MAs of randomized controlled trials (RCTs) examining the effectiveness and safety of acupuncture and related therapies on IBS.

#### 2.2.2. Types of participants.

We will include patients diagnosed with irritable bowel syndrome. The diagnostic criteria for IBS should meet the Rome III or IV criteria which include 4 types (IBS with constipation (IBC-C); IBS with diarrhea (IBS-D); IBS with mixed symptomology (IBS-M); and unclassified (IBS).^[28,29]^

#### 2.2.3. Types of interventions.

The main intervention of SRs and MAs is acupuncture. Acupuncture will not be restricted to certain types and include at least a kind of acupuncture therapy, such as manual acupuncture, electroacupuncture, auricular acupuncture, etc, or any combination of the above.

#### 2.2.4. Types of comparators.

The control groups of included SRs and MAs will be treated with conventional drugs, sham acupuncture, placebo, and no treatment.

#### 2.2.5. Types of outcome measures.

We will consider the following outcomes of 1 or more results in SRs and MAs: the overall IBS symptom, abdominal pain, distension, stool frequency and consistency, Irritable Bowel Syndrome Severity Scoring System (IBS-SSS), the 21-point numeric rating scale (NRS), the total effective rates, the cure rate, or global quality of life scores (IBS-QOL).^[30–32]^

### 2.3. Search methods for identification of studies

#### 2.3.1. Database and search.

We will design a systematic and careful retrieval scheme, and search PubMed, Embase, Cochrane Database, China National Knowledge Infrastructure (CNKI), China Science and Technology Journal Database (VIP), China Biology Medicine disc (CBMdisc), and Wanfang Database since the establishment of the database to February 2022. There will be no limits on languages.

#### 2.3.2. Search strategy.

Search terms will consist of the Medical Subject Headings (Mesh), meanwhile, synonyms and subtypes of these terms will be searched, including “Irritable bowel syndrome” (“Syndrome, Irritable Bowel”, “Colon, Irritable”, etc), “Acupuncture” (manual acupuncture, electroacupuncture, auricular acupuncture, etc), “meta-analysis” “systematic review”. These terms will be searched in combination and the grey literature will be searched as well to ensure the integrity of the search. The retrieval formula of PubMed database is presented in Table S1, Supplemental Digital Content, http://links.lww.com/MD/G694.

#### 2.3.3. Screening and selection.

**Figure 1.**
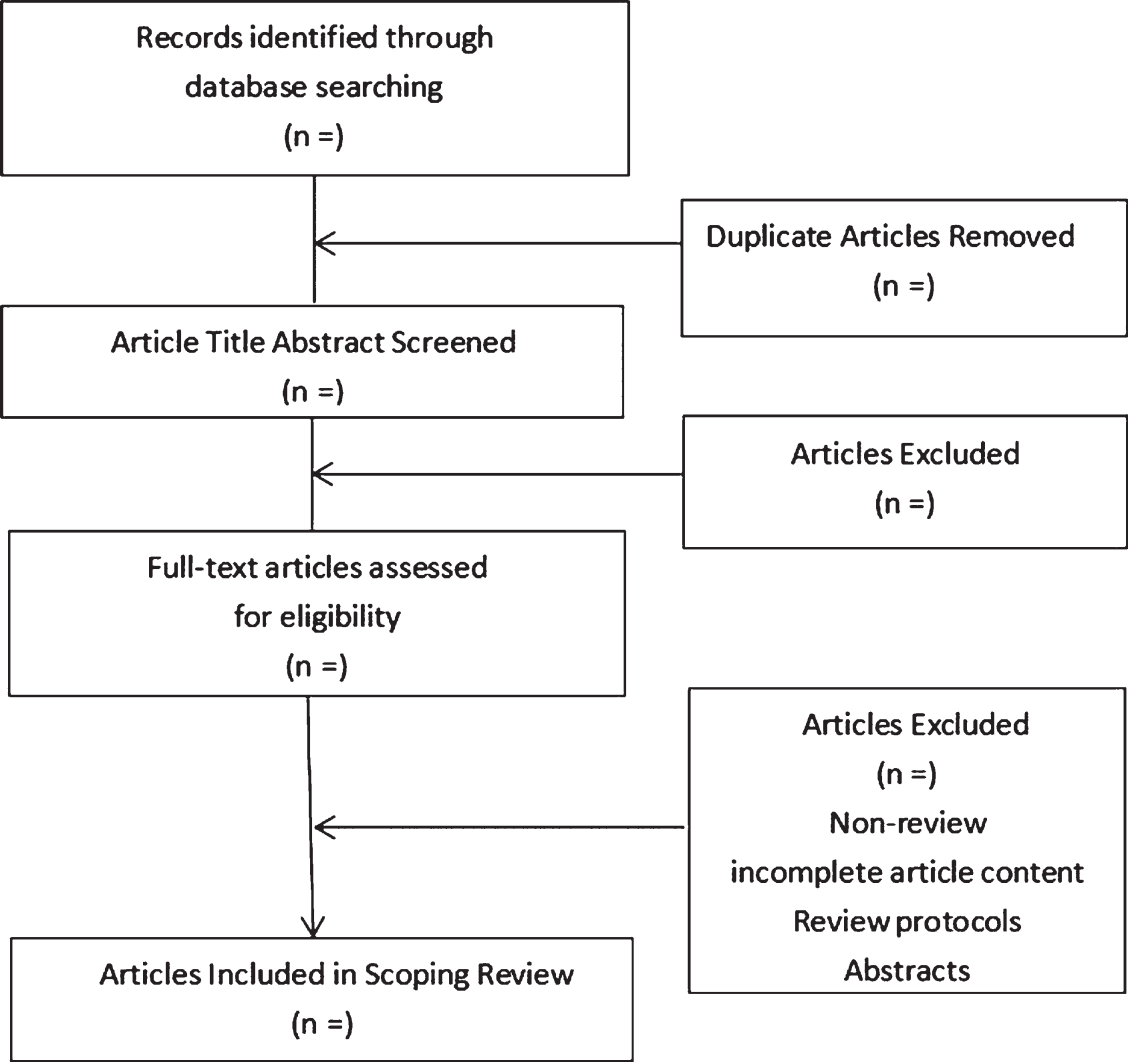
Flow diagram for study inclusion.

The data for each relevant publication will be imported into reference software (EndNote X9). Before the initial screening, the same program will be used to automatically delete any duplicate papers. The 2 researchers (LYC and PSK) will initially review the titles and abstracts of the references after deletion of duplicates, then retrieve the full text for further screening. Finally, they determine the articles that meet the inclusion criteria. If there are any disagreements, we will solve them by discussing them with another author (LJ) to ensure consistency of opinion. The specific selection process is presented in the flow diagram for study inclusion (Fig. 1).

#### 2.3.4. Data extraction.

From studies meeting inclusion criteria, 2 reviewers (LYC and PSK) independently extract data into predesigned quality assessment information tables of the included SRs/MAs, using Microsoft Excel (Microsoft, Redmont, Washington, USA). Finally, cross information to ensure the accuracy of data extraction. Data extraction will include: identifying information: publication date, first author, nationality; participants: irritable bowel syndrome and its 4 subtypes; general data: study type, sample size, and database selection; intervention measures: acupuncture and related methods were used in the experimental group, and intervention methods were used in the controlled group; research results: outcome measurement indexes and main results; methodology: data extraction method, method quality assessment, and report quality assessment; main conclusions and discussion.

### 2.4. Critical appraisal

#### 2.4.1. Quality of methodology assessment.

Two researchers (LSZ and LFY) will evaluate the quality of included studies by using the AMSTAR2 tool in duplicate. AMSTAR2 scale^[33]^ contains 16 items, each item is described by “yes” and “no”, and some items can be described by “partial yes”. Items 2, 4, 7, 9, 11, 13, and 15 are key items. If ≤1 non-key item is missing and no key item is missing, the methodological quality is high; If >1 non-key item is missing and no key item is missing, the methodological quality is medium; If 1 key item is missing, the methodological quality is low; If >1 key item is missing, the methodological quality is very low.

#### 2.4.2. Quality of report assessment.

The report quality is evaluated by PRISMA statement,^[34]^ which consists of a 27-item checklist and a 4-phase flow diagram. PRISMA includes items that are considered essential for transparent reporting of systematic reviews and meta-analyses. The included research is scored according to each item with a total score of 27 points, with the highest score was 27/27(100%) and the lowest being 0/27(0%). If the included research is fully reported, the item is recorded as 1 point, and 0 represents the lack of adequate reporting. If the score of PRISMA scale is less than 15, it is considered that the quality of the included research report has relatively serious information defects. If the score is 15 to 21, it can be considered that the quality of the included research report has certain defects, and if the score is more than 21, it can be considered the quality of the included research report. Evidence is graded on 4 levels: high, moderate, low, and extremely low.

#### 2.4.3. Evidence quality evaluation.

We will use the Grading of Recommendations, Assessment, Development, and Evaluation system to evaluate the quality of the overall evidence.^[35]^ The purpose of this approach is to evaluate the quality of evidence for each of the study's outcome indicators. Research limitations, inconsistencies in results, confusion regarding the directness of evidence, imprecise or insufficient data, and a high risk of bias are all issues that can lower the quality of evidence. To guarantee the results’ reliability and transparency, the 2 authors (LYC and PSK) will analyze the evidence independently and describe the degrading or upgrading factors that impact the quality of the evidence. The evidence will be classified into 4 grades: high, moderate, low, and very low.

#### 2.4.4. Assessment of risk of bias.

Two researchers (LFY and LSZ) will independently use the Risk of Bias in Systematic Reviews (ROBIS) tool^[36]^ to access the risk of bias of the included studies. The 3 stages of the ROBIS tool are as follows: Stage 1 assesses relevance (not applicable to this study), stage 2 identifies 4 domains, including study eligibility requirements, study discovery and selection, data collection and study assessment, and synthesis and conclusions, and stage 3 judges the risk of bias. Risk of bias is categorized as “low”, “high”, or “unclear”. If there is any disagreement, the item will be reviewed carefully again by another author (LJ) to ensure the accuracy of the results.

### 2.5. Synthesis of results

The key elements of the review available include the number of RCT included in SRs/MAs, total sample size, intervention, control, and outcome measurements. The data extracted from the study will be presented in the form of tables and pictures to facilitate our summary. The quality of the included SRs and MAs will be assessed using the Assessment of Multiple Systematic Reviews-2 for methodological quality assessment and PRISMA for reporting quality assessment. The Grading of Recommendations, Assessment, Development, and Evaluation will be used for evidence quality evaluation and ROBIS will be used to assess of risk of bias. The results will be organized in detail in tabular form. We will provide a narrative synthesis of the findings from the included SRs and MAs, as required by the Preferred Reporting Items for Systematic Reviews and Meta-Analyses Extension for Scoping Reviews (PRISMA-ScR).

## 3. Discussion

Irritable bowel syndrome is a common gastrointestinal disease with unsatisfactory clinical efficacy. The current drugs for IBS treatment cannot completely alleviate the symptoms of the patient's illness and may cause some adverse effects. Acupuncture, as an effective technique of TCM, is beneficial as an alternative treatment for irritable bowel syndrome. With the deepening and progress of evidence-based medicine, relevant SRs and MAs have been published continuously. SRs and MAs are at the top of the pyramid of evidence-based medicine, describing the validity and importance of evidence-based medicine.^[37]^ However, the results of these studies are of mixed quality and low reliability, leading to confusion in clinical decision-making. As a result, conducting a scoping review to synthetically assess the quality of these SRs/MAs is vital to offer available references for the clinical operations of acupuncture for IBS, as well as to improve the efficacy of evidence search and use.

Scoping review is a research approach for gathering and analyzing SRs and MAs linked to a specific clinical problem to obtain synthesis evidence.^[38]^ It possesses a high level of evidence, as well as the timeliness and practicality of resolving clinical issues. Therefore, the results of this scoping review will ascertain the authenticity of current evidence of irritable bowel syndrome with acupuncture, help design future SRs/MAs, and determine research directions to address gaps in existing knowledge. However, the study also has several anticipated limitations and challenges including low quality of original research, difficulties in synthesizing and grading evidence from different types of clinical studies, and frequency of intervention will lead to some bias and eventually affect the credibility of this study.

## Acknowledgments

The authors thank Dr. Yan-Ji Zhang from Hubei University of Traditional Chinese Medicine for their suggestions on the design of this review.

## Author contributions

Yachen Li is the first author and Sike Peng is the joint first author. Yachen Li and Sike Peng are the study guarantors. All authors have read and approved the publication of the protocol.

Conceptualization: Yachen Li, Sike Peng, Fangyuan Liang, Suzhen Liu, Jia Li.

Data curation: Sike Peng, Fangyuan Liang, Suzhen Liu

Formal analysis: Yachen Li, Jia Li.

Investigation: Yachen Li, Sike Peng, Fangyuan Liang, Jia Li.

Methodology: Yachen Li, Suzhen Liu.

Software: Fangyuan Liang, Suzhen Liu.

Supervision: Jia Li.

Writing – original draft: Yachen Li, Sike Peng.

Writing – review & editing: Jia Li.

## Supplementary Material


