# Supplementary material for: Effectiveness of acupuncture for irritable bowel syndrome: Protocol for a scoping review of systematic reviews and meta-analyses
Source: Medicine (Baltimore). 2022 Jul 22;101(29):e29218. doi: 10.1097/MD.0000000000029218 (PMC9302279; doi:10.1097/MD.0000000000029218)
Supplement: Supplementary file 1 [file medi-101-e29218-s001.docx]

**Table 1 retrieval formula in PubMed database**
